# ω-3PUFA supplementation ameliorates adipose tissue inflammation and insulin-stimulated glucose disposal in subjects with obesity: a potential role for apolipoprotein E

**DOI:** 10.1038/s41366-021-00801-w

**Published:** 2021-03-22

**Authors:** James D. Hernandez, Ting Li, Cassandra M. Rau, William E. LeSuer, Panwen Wang, Dawn K. Coletta, James A. Madura, Elizabeth A. Jacobsen, Eleanna De Filippis

**Affiliations:** 1 Division of Endocrinology and Metabolism, Mayo Clinic Arizona, Scottsdale, AZ USA; 2 Arizona State University, Barrett, The Honors College, Tempe, AZ USA; 3 Division of Allergy and Immunology, Mayo Clinic Arizona, Scottsdale, AZ USA; 4 Department of Biostatistics, Mayo Clinic Arizona, Scottsdale, AZ USA; 5 Department of Medicine, College of Medicine, University of Arizona, Tucson, AZ USA; 6 Division of General Surgery, Mayo Clinic Arizona, Phoenix, AZ USA

**Keywords:** Metabolic syndrome, Obesity, Immunology, Nutrition

## Abstract

**Background:**

Long chain omega-3 polyunsaturated fatty acids (ω-3PUFA) supplementation in animal models of diet-induced obesity has consistently shown to improve insulin sensitivity. The same is not always reported in human studies with insulin resistant (IR) subjects with obesity.

**Objective:**

We studied whether high-dose ω-3PUFA supplementation for 3 months improves insulin sensitivity and adipose tissue (AT) inflammation in IR subjects with obesity.

**Methods:**

Thirteen subjects (BMI = 39.3 ± 1.6 kg/m^2^) underwent 80 mU/m^2^·min euglycemic-hyperinsulinemic clamp with subcutaneous (Sc) AT biopsy before and after 3 months of ω-3PUFA (DHA and EPA, 4 g/daily) supplementation. Cytoadipokine plasma profiles were assessed before and after ω-3PUFA. AT-specific inflammatory gene expression was evaluated on Sc fat biopsies. Microarray analysis was performed on the fat biopsies collected during the program.

**Results:**

Palmitic and stearic acid plasma levels were significantly reduced (*P* < 0.05) after ω-3PUFA. Gene expression of pro-inflammatory markers and adipokines were improved after ω-3PUFA (*P* < 0.05). Systemic inflammation was decreased after ω-3PUFA, as shown by cytokine assessment (*P* < 0.05). These changes were associated with a 25% increase in insulin-stimulated glucose disposal (4.7 ± 0.6 mg/kg ffm•min vs. 5.9 ± 0.9 mg/kg ffm•min) despite no change in body weight. Microarray analysis identified 53 probe sets significantly altered post- ω-3PUFA, with Apolipoprotein E (APOE) being one of the most upregulated genes.

**Conclusion:**

High dose of long chain ω-3PUFA supplementation modulates significant changes in plasma fatty acid profile, AT, and systemic inflammation. These findings are associated with significant improvement of insulin-stimulated glucose disposal. Unbiased microarray analysis of Sc fat biopsy identified APOE as among the most differentially regulated gene after ω-3PUFA supplementation. We speculate that ω-3PUFA increases macrophage-derived APOE mRNA levels with anti-inflammatory properties.

## Introduction

Obesity is a worldwide public health issue [1], which causes a chronic, low-grade inflammatory state [2, 3]. It predisposes to the development of cardiovascular disease [4], insulin resistance [5] and type 2 diabetes mellitus [6], all maladies with high morbidity and mortality rates [7].

It is increasingly clear that adipose tissue (AT) is not simply a passive store of excess fat [8], but is an endocrine organ that plays key roles in physiologic and pathologic processes [8]. In health, AT mobilizes its stores as free fatty acids (FFA) which is released during fasting states as fuel for lean tissues [8]. In obesity, there is an increased number of pro-inflammatory macrophages within the AT (up to 20–30% of AT-resident immune cells) [9]. Pro-inflammatory macrophages, referred to as M1, synthetize and release inflammatory cytokines, such as TNFα, IL-1, and IL-6 [10, 11]. Excess of circulating FFAs or elevated levels of pro-inflammatory cytokines impair insulin signaling and promote the establishment of insulin resistance [12, 13]. Decreasing AT inflammation could represent a therapeutic approach for the amelioration of insulin resistance in subjects with obesity.

The polyunsaturated fatty acid omega-3 (ω-3PUFA) is reported to have an anti-inflammatory effect [14, 15]. Regular consumption of fish and/or oral supplementation with fish oil derivatives are important dietary sources of ω-3PUFA [15]. Many studies have evaluated the effect of fish oil supplementation on the improvement of insulin resistance in diet-induced obesity (DIO) animal models [16]. Many mechanisms are linked to such metabolic improvement [16]. However, the ability for ω-3PUFA to effectively improve insulin sensitivity in human studies remains inconsistent [17].

Recently, Antigoni et al. highlighted possible reasons for the discordance observed in human studies and the lack of translation from animal studies, citing varying dosage, source of fish oil, duration of intervention, and the methods used to assess insulin sensitivity as plausible reasons for this discrepancy [17]. Another current publication evaluating multiple ω-3PUFA formulations commercially available revealed much variation among the products, highlighting the importance of third-party evaluation even on products meeting FDA standards [18]. Studies with order of magnitude differences in dosing ranges, from milligrams to grams daily, have been published. Combined with an unknown purity of the supplement, many confounding errors may result.

A recent review [19] concluded for the beneficial effects of ω-3PUFA supplementation on CVD risks to be greatest in subjects achieving the highest blood concentration. The OMEGA-REMODEL [20] and REDUCE-IT [21] trials showed doses of 4 g/day have been successfully used as secondary prevention to decrease risk of CVD death. These trials measured improvement of a disease status. We based our study dosing strategy on these models, focusing on improvement of existing insulin resistance through high-dose supplementation, whereas animal studies have focused on preventing the onset of insulin resistance. Here we sought to determine whether 3 months of 4 g/day ω-3PUFA improves insulin sensitivity in subjects with obesity and documented evidence of insulin resistance and systemic inflammation. Our hypothesis was that high-dose ω-3PUFA supplementation, by modifying plasma fatty acid profile, would decrease systemic as well as localized AT inflammation, thereby effectively improving insulin sensitivity in humans.

## Subjects and methods

Participants were recruited via advertisement at Mayo Clinic Arizona, local newspapers, and the internet (www.clinicalconnection.com or https://clinicaltrials.gov/ct2/show/NCT02378077). Eligible participants were between the age of 18 and 65 years, non-smokers, with BMI ≥ 30 kg/m^2^, no significant weight loss for 6 months before the study enrollment. Subjects were excluded from the study if having diabetes mellitus, atopic syndromes, documented asthma, anemia, or an inborn or acquired bleeding disorder. Subjects taking glucocorticoid medications, statins, any antidiabetic medications, or anti-coagulants were also excluded. Subjects on no ω-3PUFA supplementation and consuming fish in their diet less than 3 times a week were eligible for the study. The study was reviewed and approved by the Mayo Clinic IRB and was registered at clinicaltrial.gov (NCT02378077). All studies were conducted in the Clinical Studies Infusion Unit (CSIU) at the Mayo Clinic Arizona in accordance with ethical standards of the institution.

Upon obtaining informed, written consent, subjects received a medical history intake, physical examination, and a complete chemistry panel. Body composition was obtained using BIA 310 Bioimpedance Analyzer (Biodynamics Corporation, Shoreline, WA, USA). Next, a 75 g oral glucose tolerance test (OGTT) was conducted to confirm normal glucose tolerance by ADA criteria. Insulin resistance was determined using the composite index also referred to as Matsuda index [22] where a value below 3 identifies subjects with insulin resistance [23]. Subjects defined as insulin resistant underwent further evaluation through euglycemic-hyperinsulinemic clamp to better characterize changes of insulin sensitivity in response to fish oil supplementation.

### Euglycemic-hyperinsulinemic clamp

Before and after 3 months of ω-3PUFA supplementation, after an overnight fast, subjects underwent a euglycemic-hyperinsulinemic clamp (80 mU/m^2^·min) to assess insulin sensitivity response. Briefly, a retrograde catheter was inserted into a dorsal hand vein, and the hand placed in a heated box (55 °C) for collection of arterialized blood. Venous catheter in the contralateral arm was used for infusion of glucose and insulin. Two hours prior to insulin infusion; a primed infusion of 6,6 di-deuterated glucose was begun to determine the basal rate of glucose metabolism. After 2 h, a primed continuous infusion of insulin was started. The constant infusion of deuterated glucose was discontinued at time 15 min after the start of the insulin infusion, and a variable infusion of 20% dextrose that was enriched with 6,6 di-deuterated glucose was used to maintain euglycemia (∼90 mg/dL) and a constant enrichment of the tracer. Plasma glucose levels were determined by the glucose oxidase method on an YSI 2300 STAT plus (YSI INC., Yellow Springs, OH, USA). At the end of the study, all hormonal infusions were turned off, dextrose infusion was slowly weaned while subjects received a meal. After reaching steady state off of any dextrose infusion, subjects were dismissed [24]. All subjects were sedentary and were asked to refrain from exercise for at least 48 h before any study procedure.

### Fat biopsy

On a separate day, during fasting, a Sc fat biopsy in the lower abdominal area between the navel and the pubic area was performed in sterile fashion, as previously described [25]. A range of 3–5 g was isolated in a single extraction. Adipocytes were isolated from 2 g of the AT biopsy as previously described [26, 27]. After last centrifugation, the top oil layer represented floating adipocytes which was collected then flash frozen for future mRNA evaluation. The Sc AT biopsy was repeated after the completion of 3 months of ω-3PUFA supplementation.

### Immunohistochemistry

Immunohistochemistry was performed by the Pathology Research Core at Mayo Clinic, Rochester, MN as previously described [25]. The antibody selection was based on a previous study conducted at the same institution [28], CD68 as a marker of total macrophage, CD14 as a pro-inflammatory (M1) macrophage marker, CD206 as an anti-inflammatory (M2) macrophage marker.

### Immunohistochemistry (IHC) quantification

The stained tissue sections were scanned at ×20 with an Aperio ScanScope AT Turbo slide scanner (Leica Biosystems Inc., Buffalo Grove, IL). Five randomly selected images per slide were taken at ×20 magnification. Two independent observers counted positively stained macrophages and total adipocytes for each field (measuring 500 × 500 microns, five per slides) using a program named AM Counter [28]. Stained cells displaying the known morphological characteristics of macrophages were counted as adipose tissue macrophages (ATM). From this, we determined the ratio of ATM to adipocytes per field of view. All slides were marked with a code rather than the sample identity to ensure the independent observers were blinded to the participants.

### ω-3PUFA fish oil supplementation

Nature Made Burp-less Fish Oil was chosen based on several criteria; holding a high percentage of the retail consumer market making it readily available, adherence to FDA manufacturing standards, low reported side effect profile, and recommendation by in house Mayo Clinic pharmacy. Study subjects were dispensed a 1-month supply (120 g) of Nature Made Burp-less Fish Oil capsules, 500 mg per capsule (Nature Made, West Hills, CA, USA) (Lot# 2217828) to be ingested at 4 g/day with meals. Subjects ingested four capsules in the morning with breakfast and four capsules in the evening with dinner.

### Study follow-up visits

Subjects met with a study coordinator on a monthly basis. During this visit, any side effects were discussed, remaining fish oil capsules were returned and counted, and a new monthly supply of fish oil was distributed. The subjects were also questioned on any changes in weight, activity levels, and dietary fish oil consumption. Lastly, a fasting blood sample was collected for plasma FFA analysis.

### Assessment of ω-3PUFA content in fish oil

Quality control on the ω-3PUFA content was completed independently by CromaDex Inc., Irvine, CA, USA by gas chromatography as per their protocol.

### Real-time PCR

Total RNA was isolated from intact AT and isolated adipocytes using the RNeasy Plus Mini Kit (Qiagen Germantown, MD, USA) according to the manufacturer’s instruction. 1 μg total RNA was converted to cDNA using High-Capacity cDNA Reverse Transcription Kit as described by the manufacturer (Applied Biosystems Waltham, MA, USA). Real-time PCR (RT-PCR) analysis was performed using CFX384 Touch RT-PCR Detection System (Bio-Rad Hercules, California, USA) with specific primers (Table S1) and iTaq™ Universal SYBR^®^ Green Supermix (Bio-Rad). β-actin was our internal control gene.

### Microarray

RNA from isolated adipocytes was prepared for hybridization to Affymetrix (Santa Clara, CA, USA) HG-U133 arrays according to the manufacturer’s instruction. Microarray data were generated blindly using a coded ID.

### Microarray data expression and analysis

The cell intensity files were generated from the stored images that contain a single intensity value for each probe cell on the array. After normalization, the expression values obtained were submitted for analysis with linear models of microarray data (LIMMA v3.38.3). To correct for multiple testing, *P* values were adjusted using the method of Benjamin and Hochberg. Genes with false discovery rate <5% and fold change >1.5 were classified as significantly differentially expressed. Gene function annotation and enrichment analysis were performed using Database for Annotation, Visualization, and Integrated Discovery (DAVID) [29]. We used Enrichr [30] and GSEA [31] for pathway enrichment analysis.

### Plasma and serum analysis

The screening laboratory tests and metabolic panel were performed by the Biospecimens Accessioning and Processing Core at the Mayo Clinic Arizona. Fasting plasma glucose was measured by the YSI 2300 STAT plus Glucose and Lactate Analyzer (YSI INC., Yellow Sprigs, OH, USA) in the CSIU. Serum insulin was measured at the Immunochemical Core Laboratory at the Mayo Clinic Rochester. The 6,6-^2^H2 glucose enrichment data from the euglycemic-hyperinsulinemic clamp was measured blindly at the Center for Clinical and Translational Science Metabolomics Core at the Mayo Clinic Rochester. Human plasma IL-6 and adiponectin levels were measured by ELISA kits according to the manufacturer’s instructions (R&D Systems Minneapolis, MN, USA). Human plasma C-reactive protein (CRP) levels were measured by ELISA kit according to the manufacturer’s instructions (ThermoFisher Invitrogen, MA, USA). Human Cytokine Array Pro-inflammatory Focused 13-plex (HDF13) was performed to measure cytokines in plasma (Eve Technologies Corporation Alberta, Canada). APOE and APOC1 plasma levels were assessed by ELISA according to the manufacturer’s instructions (ThermoFisher Invitrogen, MA, USA).

### Plasma free fatty acids species profiling

Fasting plasma samples were sent to the Vanderbilt University Medical Center Lipid Core (Nashville, TN, USA) for blind evaluation of free fatty acids profiling by HPLC analysis.

### Power and statistical analysis

Spencer et al. [32] described paired mean difference in MCP-1 plasma levels for obese subjects on fish oil of ~10 pg/mL with an estimated standard deviation of 10.5. Based on these data, it was calculated that the total number of subjects recruited for the study would have 80% power to detect an effect size of 0.9 with a significance level of 0.05 by Wilcoxon signed-rank test. Prior to the initiation of biostatistical analysis, the continuous variables were checked for normal distribution using the D’Agostino-Person omnibus test and the Shapiro–Wilk test [33]. After confirmation of normally distributed variables, statistical significance of the difference between means before and after fish oil supplementation was determined using paired Student’s *t*-test. Kruskal–Wallis test was used for the analysis of serum FFA changes during the study (Table 1). *P* ≤ 0.05 was considered statistically significant. Data were expressed as a means ± SE. All plasma cytokines, serum insulin, and plasma FFA levels were blindly quantified by a third party.

**Table 1:** Characteristic of subjects and FFA plasma levels before and after ω-3PUFA supplementation.

	Pre-FO	1 month	2 months	Post-FO	*P* value vs. Pre-FO
Total number of subjects	13				
Sex (F/M)	10/3				
Age (years)	40.7 ± 2.4				
C-reactive protein (CRP) (mg/L)	7.2 ± 0.5				
Body mass index (BMI) (kg/m^2^)	39.3 ± 1.6			39.5 ± 1.6	*P* = 0.923
Systolic blood pressure (SDP) (mmHg)	125.2 ± 2.5			113.9 ± 1.9	*P* < 0.01
Diastolic blood pressure (DBP) (mmHg)	76.5 ± 1.7			73.0 ± 1.7	*P* = 0.220
Pulse (bpm)	72.2 ± 4.1			70.5 ± 2.1	*P* = 0.743
% Body fat	42.2 ± 1.8			41.7 ± 2.0	*P* = 0.874
Weight (kg)	106.7 ± 6.3			107.5 ± 6.6	*P* = 0.933
Fasting glucose (mmol/L)	5.2 ± 0.1			5.5 ± 0.1	*P* = 0.522
A1C (%)	5.3 ± 0.1			5.4 ± 0.1	*P* = 0.455
Triglycerides (mmol/L)	1.2 ± 0.2			1.2 ± 0.2	*P* = 0.906
Cholesterol (mmol/L)	4.6 ± 0.2			4.9 ± 0.2	*P* = 0.340
High-density lipoproteins (HDL) (mmol/L)	1.2 ± 0.1			1.3 ± 0.1	*P* = 0.950
Low-density lipoproteins (LDL) calculated (mmol/L)	2.8 ± 0.2			3.1 ± 0.2	*P* = 0.286
Docosahexaenoic acid (DHA) (mM)	0.002 ± 0.001	0.004 ± 0.001	0.005 ± 0.001	0.005 ± 0.001	*P* < 0.01
Eicosapentaenoic acid (EPA) (mM)	0.000 ± 0.000	0.000 ± 0.000	0.001 ± 0.000	0.001 ± 0.000	*P* < 0.05
Total FFAs (mM)	0.719 ± 0.055	0.408 ± 0.045	0.404 ± 0.029	0.578 ± 0.061	*P* < 0.001
Palmitic acid (mM)	0.165 ± 0.011	0.098 ± 0.008	0.099 ± 0.007	0.135 ± 0.012	*P* < 0.001
Stearic acid (mM)	0.066 ± 0.005	0.045 ± 0.003	0.046 ± 0.003	0.060 ± 0.004	*P* < 0.05
Oleic acid (mM)	0.280 ± 0.023	0.149 ± 0.020	0.143 ± 0.012	0.219 ± 0.027	*P* < 0.001
Linoleic acid (mM)	0.131 ± 0.011	0.073 ± 0.009	0.073 ± 0.006	0.100 ± 0.011	*P* < 0.001

## Results

### Study population and intervention

Twenty-eight subjects were screened, four subjects failed because of: anemia [1], diagnosis of diabetes mellitus [2], and excessive weight loss [1]. In addition, eight subjects did not adhere to study schedule and three did not tolerate fat biopsy leading to 13 subjects enrolled with completion of all visits (Fig. S1). No subjects withdrew due to side effects of fish oil. No significant changes in BMI, or fat percentage were identified after 3 months of ω-3PUFA supplementation in the studied subjects (Table 1). After ω-3PUFA supplementation, systolic blood pressure was significantly decreased in our cohort as previously described [34].

### Analytical test report for EPA, DHA, and ω-3 analysis by GC

In each capsule, the total EPA and DHA fatty acid resulted to be within the reported range at 466 mg/serving (range NLT-500 mg/serving) with a ratio of n-6: n-3 (total Omega-3 Fatty Acids 533 mg/serving: total Omega-3 Fatty Acids 55.3 mg/serving) equals around 1:10 (Table S2).

### Plasma FFA levels analysis

ω-3PUFA capsules were well tolerated. Participants did not complain of any nausea, diarrhea, change in bowel habit, or increased bleeding time. Adherence to supplement was assessed by counting remaining capsules at the follow-up visits (not shown) and by plasma level determination of ω-3PUFA fatty acyl moieties at Pre-FO and at the end of study. We found both DHA and EPA plasma levels to be significantly increased (Table 1).

Over the course of 3 months supplementation, we observed a significant reduction in total FFA from Pre-FO to the subsequent visits (Table 1). Of all specific fatty acyl moieties, we demonstrated a reduction by 40% for palmitic acid and 30% for stearic acid. Though we found decreases in oleic acid and linoleic acid (Table 1), these levels were comparable to those found in plasma from lean subjects (BMI 22.1 ± 0.4 kg/m^2^) of another study (data not shown).

### Plasma inflammatory cytokines and adipokines

**Fig. 1:**
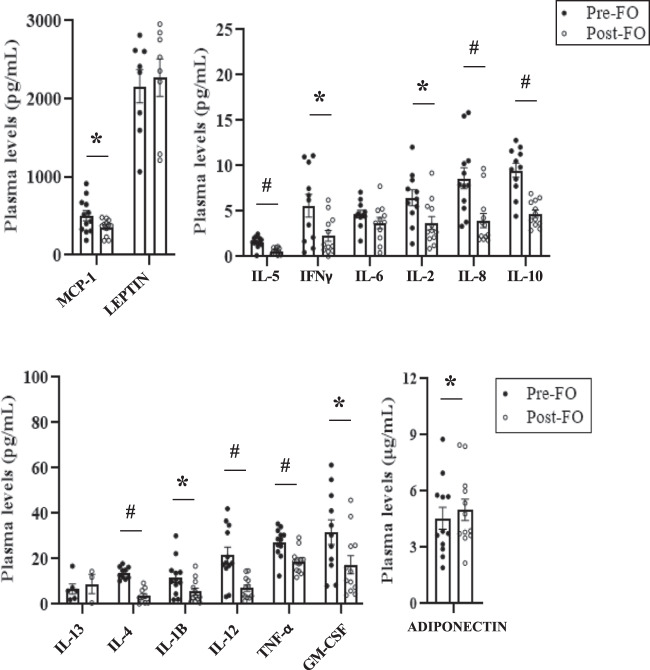
Changes in adipokine and cytokines in plasma after omega-3 polyunsaturated fatty acids (ω-3PUFA) supplementation. Plasma cytokines levels were significantly changed after ω-3PUFA supplementation compared with Pre-FO. Monocyte Chemoattractant Protein-1 (MCP-1), tumor necrosis factor-α (TNF-α), interleukin-1β (IL-1β), interleukin-6 (IL-6), interleukin-4 (IL-4), interleukin-13 (IL-13), interleukin-10 (IL-10), interleukin-5 (IL-5), interleukin-2 (IL-2), interleukin-8 (IL-8), interleukin-12 (IL-12), interferon-γ (IFN-γ), and granulocyte-macrophage colony-stimulating factor (GM-CSF). Multiplex cytokines array was completed as described in “Subjects and methods” section. Paired *t*-test was completed for each cytokine and adipokine pre vs. post-FO. Data are expressed as means ± SE. *n* = 13 *P < 0.05 vs. pre-FO; ^*#*^*P* < 0.01 vs. pre-FO.

Prior to ω-3PUFA supplementation, plasma levels of inflammatory markers including CRP confirmed the presence of systemic inflammation in the setting of obesity and insulin resistance (Table 1 and Fig. 1). After ω-3PUFA supplementation, plasma levels of adiponectin were significantly increased, while leptin was found to be unchanged (data not shown). We demonstrated that plasma levels of pro-inflammatory cytokines MCP-1, TNF-α, IL-1B, INFγ, and GM-CSF were all significantly decreased after 3 months of FO (Fig. 1). In addition, we showed changes in other interleukins including significantly decreased IL-2, IL-8, and interestingly an unexpected decrease in IL-4 and IL-10.

### Adipose tissue inflammatory markers

**Fig. 2:**
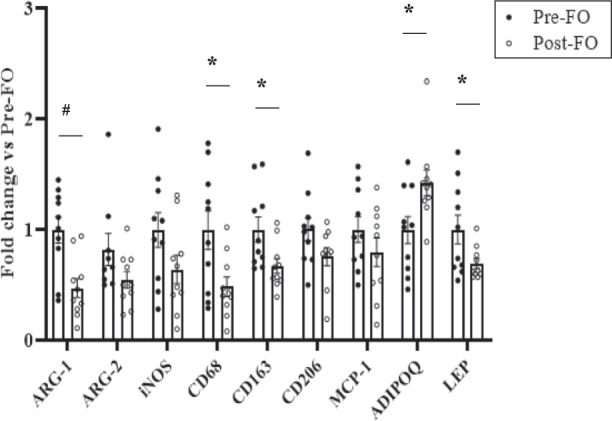
Changes in adipokines and cytokines in adipose tissue after omega-3 polyunsaturated fatty acids (ω-3PUFA) supplementation. Adipose tissue inflammatory markers and adipokines mRNA levels were significantly changed after ω-3PUFA supplementation compared with Pre-FO. Quantitative real-time PCR analysis of gene expression of arginase 1 (ARG-1), arginase 2 (ARG-2), inducible nitric oxide synthase (iNOS), cluster of differentiation 68 (CD68), cluster of differentiation 163 (CD163), cluster of differentiation 206 (CD206), monocyte chemoattractant protein-1 (MCP-1), adiponectin (Adipoq), and leptin (Lep). Paired *t*-test was completed for each cytokine and adipokine pre vs. post-FO. Data are expressed as means ± SE. *n* = 13 *P < 0.05 vs. pre-FO; ^*#*^*P* < 0.01 vs. pre-FO.

A significant decrease in pro-inflammatory macrophages markers (mRNA levels of iNOS (*P* < 0.05), CD68 (*P* < 0.05), CD163 (*P* < 0.05)) was found within the subcutaneous AT of our IR subjects with obesity (Fig. 2). After ω-3PUFA supplementation, AT mRNA levels of adiponectin were significantly increased (*P* < 0.05), while leptin was decreased (*P* < 0.05) (Fig. 2), without any change in adipocytes size (Fig. S2). We did not detect any changes in the total number of macrophages (M1 to M2 population) after fish oil supplementation (Fig. S3).

### Assessment of insulin resistance: OGTT and euglycemic-hyperinsulinemic clamp

Data collected during the OGTT were used to calculate the Matsuda index to assess for the presence of insulin resistance before initiation of ω-3PUFA supplementation. Our calculations demonstrated for the Matsuda index to be 2.4 ± 0.2, indicating that our subjects were indeed insulin resistant. This was further confirmed by the low insulin-stimulated glucose disposal rate, 4.7 ± 0.6 mg/kg ffm•min, calculated during the euglycemic-hyperinsulinemic clamp (Table 2). A rate below 5.3 mg/kg ffm•min is considered to indicate insulin resistance [35].

**Table 2:** Euglycemic-hyperinsulinemic clamp before and after ω-3PUFA supplementation.

	Pre-FO	Post-FO	*P* value vs. Pre-FO
Endogenous glucose production (EGP) (basal) (mg/kg ffm•min)	2.9 ± 0.1	3.0 ± 0.1	*P* = 0.524
EGP during insulin infusion	0.0 ± 0.3	0.0 ± 0.1	*P* = 0.819
(mg/kg ffm•min)			
Glucose disposal	4.7 ± 0.6	5.9 ± 0.9	*P* < 0.05
(mg/kg ffm•min)			
Plasma insulin level (basal)	16.2 ± 4.5	19.9 ± 8.0	*P* = 0.701
(µIU/ml)			
Plasma insulin level (90–120 min insulin infusion)	190.2 ± 17.3	203.9 ± 12.0	*P* = 0.538
(µIU/ml)			

Endogenous glucose production, which is suppressed during the euglycemic clamp, was found to be similar both before and after ω-3PUFA (Table 2). After 3 months of ω-3PUFA supplementation, insulin-stimulated glucose disposal was increased by 25% (Pre-FO 4.7 ± 0.6 mg/kg ffm•min vs. post-FO 5.9 ± 0.9 mg/kg ffm•min) with no change in mean insulin infused levels (Table 2).

### Adipose tissue transcriptomic analyses

We identified the expression of 34 probes which were significantly increased (Fold change > 1.50, *P* < 0.05, uncorrected), and 19 which were significantly decreased (Fold change < 0.67, *P* < 0.05, uncorrected) when we compared post-FO with pre-FO fat biopsy samples. Out of those 53 probes, we successfully mapped them to 44 genes. We then performed GOTERMs pathway enrichment analysis of those differentially expressed genes. In the top 5 of the enriched terms were the gene pathways for Inflammatory Response, Collagen Catabolic Process, and Extracellular Matrix Disassembly (Table 3). Of particular interest, APOE and APOC1 were present in all of the above pathways.

**Table 3:** Top 26 enriched pathways by DAVID functional analysis tool.

No. of rank	Term name	Genes	*P* value
1	GO:0006954~inflammatory response	S1PR3, TNFAIP6, TNFRSF11A, CHI3L1, SPP1	0.003
2	GO:0030574~collagen catabolic process	MMP9, MMP7, COL8A2	0.004
3	GO:0034447~very-low-density lipoprotein particle clearance	APOE, APOC1	0.005
4	GO:0022617~extracellular matrix disassembly	MMP9, MMP7, SPP1	0.006
5	GO:0050728~negative regulation of inflammatory response	TNFAIP6, APOE, ACP5	0.007
6	GO:0001503~ossification	TNFRSF11A, MMP9, SPP1	0.007
7	GO:0034382~chylomicron remnant clearance	APOE, APOC1	0.009
8	GO:0006032~chitin catabolic process	CHI3L1, CHIT1	0.011
9	GO:0051549~positive regulation of keratinocyte migration	MMP9, HAS2	0.011
10	GO:0071356~cellular response to tumor necrosis factor	CHI3L1, HAS2, DCSTAMP	0.012
11	GO:0010873~positive regulation of cholesterol esterification	APOE, APOC1	0.014
12	GO:0034374~low-density lipoprotein particle remodeling	APOE, PLA2G7	0.017
13	GO:0045780~positive regulation of bone resorption	DCSTAMP, SPP1	0.020
14	GO:0033700~phospholipid efflux	APOE, APOC1	0.021
15	GO:0034375~high-density lipoprotein particle remodeling	APOE, APOC1	0.023
16	GO:0048168~regulation of neuronal synaptic plasticity	EGR2, APOE	0.024
17	GO:0044849~estrous cycle	MMP7, HAS2	0.026
18	GO:0030316~osteoclast differentiation	TNFRSF11A, DCSTAMP	0.035
19	GO:0034612~response to tumor necrosis factor	TNFRSF11A, CHI3L1	0.038
20	GO:0033344~cholesterol efflux	APOE, APOC1	0.038
21	GO:0060349~bone morphogenesis	ACP5, HAS2	0.041
22	GO:0070555~response to interleukin-1	TNFRSF11A, CHI3L1	0.050
23	GO:0006641~triglyceride metabolic process	APOE, APOC1	0.053
24	GO:0042157~lipoprotein metabolic process	APOE, APOC1	0.057
25	GO:0007566~embryo implantation	MMP9, SPP1	0.063
26	GO:0034097~response to cytokine	TNFRSF11A, ACP5	0.078

**Fig. 3:**
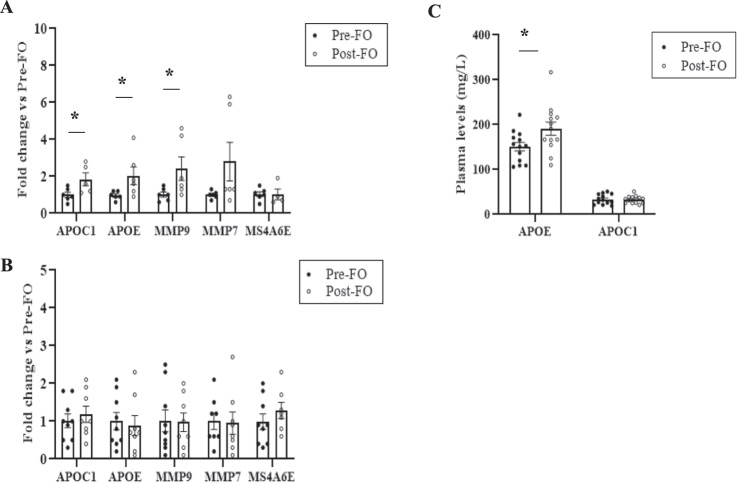
Adipose tissue mRNA expression of selected genes and plasma protein levels before and after omega-3 polyunsaturated fatty acids (ω-3PUFA) supplementation. Several mRNA gene levels were tested on Sc fat biopsy (whole tissue) (**A**) or pure adipocytes fraction after collagenase from Sc fat biopsy (**B**) to validate whole fat microarray analysis. As shown, several genes are significantly upregulated post-FO in whole adipose tissue (**A**), but not in the adipocyte sub-fraction (**B**). **C** Plasma levels of APOE and APOC1 were determined by ELISA. APOE, but not APOC1, plasma levels were significantly elevated post-FO. Apolipoprotein C1 (APOC1), membrane spanning 4-domains A6E (MS4A6E), matrix metallopeptidase 9 (MMP9), apolipoprotein E (APOE), matrix metalloproteinase 7 (MMP7). Paired *t*-test was used to compare pre vs. post mRNA changes (*n* = 7) and pre vs. post plasma levels (*n* = 13). Data are expressed as means ± SE. *P < 0.05 vs. pre-FO.

Microarray data analysis validation showed significantly elevated APOE, APOC1, MMP9, and MMP7 (*P* < 0.05) (Fig. 3a) gene expression in AT after ω-3PUFA. We specifically focused on changes in APOE mRNA expression as they were reflected by increased APOE protein plasma levels after ω-3PUFA supplementation (Fig. 3c), whereas APOC1 protein levels were not elevated (data not shown). We then analyzed the expression level of the gene in a sample of purified adipocytes from subcutaneous fat biopsy. We were unable to identify an increase in gene expression on the pure adipocyte sub-fraction (Fig. 3b). Therefore, we speculate that the increase in APOE mRNA expression results from an increased gene expression in the stromal vascular fraction which includes resident macrophages.

## Discussion

Our results highlight the potential of high-dose ω-3PUFA supplementation as a mediator of increased insulin sensitivity in subjects with obesity, insulin resistance and elevated biomarkers of plasma and AT inflammation. We demonstrate that 3 months of high dose (4 g/day), supplier and lot number controlled, ω-3PUFA supplementation is sufficient to improve plasma FFA profile and inflammatory status. These observations were associated with a 25% improvement in insulin-stimulated glucose uptake in our IR subjects with obesity. Furthermore, AT analysis by microarray highlighted an increased expression of APOE mRNA in subcutaneous fat with a corresponding elevation in plasma protein levels. It appears however that this increase was not mediated by adipocytes, therefore we speculate for AT macrophage to be the source of this increase in APOE.

After ω-3PUFA, we found a significant reduction in plasma levels of palmitic acid, a fatty acid moiety previously associated with decreased levels of adiponectin [36] and increased markers of inflammation [13]. The plasma composition of saturated fatty acids impacts both the circulating inflammatory state and that of the AT [13, 37, 38]. Our data revealed significantly reduced plasma inflammatory cytokines and decreased mRNA levels of pro-inflammatory markers of macrophages. In agreement with our study, Spencer et al. described a significant decrease in MCP-1 in both plasma and AT of insulin resistant subjects supplemented with 4 g/daily of fish oil for 12 weeks [32]. However, they reported no change in insulin sensitivity, circulating levels of adipokines, nor any cytokines other than MCP-1. A potential explanation for such discrepancy in our study may be related to the observed improvement of adiponectin, a known mediator of insulin sensitivity. In addition, we identified significant changes in the circulating cytokine profile after ω-3PUFA supplementation. While historically IL-10 has been considered having anti-inflammatory properties, recent data from animal [39] and in vitro human [40] studies have disputed this understanding. In the animal study mentioned, ablation of IL-10 protects against DIO, improves insulin sensitivity, and increases browning of white ATs. Similarly, in a cohort of patients with obesity, IL-10 positivity correlates with indices of insulin resistance. Analysis of stromal vascular fraction isolated from human subcutaneous and visceral AT demonstrated for IL-10 to be enriched in pro-inflammatory macrophages. In our study, we suggest for the reduction of IL-10 plasma level to be linked to the anti-inflammatory effect of FO. The decrease in IL-4 shown in our data may be a result of IL-4 having been described as a pleiotropic cytokine with quite differing functions in different tissues [41]. In AT, IL-4 is derived from adipocytes and various immune cell populations including Th2 lymphocytes, mast cells and eosinophils. A recent in vitro study [42] using a murine cell system demonstrated for IL-4 to inhibit adipogenesis and promote lipolysis, which were not assessed in our study. Furthermore, our total FFA level is decreased suggesting no increase in lipolysis which may fit with a decreased IL-4 plasma concentration. Additional studies are needed to explore changes in immune cell populations beyond macrophage which may be affected by fish oil supplementation and what role those changes can have in the secretion of IL-4.

Previous human studies have failed to show improvement of insulin sensitivity after fish oil supplementation [17]. Lalia et al. [43] used fish oil doses comparable to ours for a 6-month duration. Their pancreatic clamp showed no improvements in insulin-mediated AT lipolysis. We believe that the difference with our results is ascribed to the subjects’ characteristics. Our enrolled subjects were people with obesity (BMI = 39 kg/m^2^) with clear evidence of insulin resistance and inflammation. Lalia et al. [43] enrolled overweight subjects (BMI > 25 kg/m^2^) that possibly started with less inflammation and less insulin resistance than what we observed. This was indeed pointed out in the study conducted by Browning et al, who found significant changes in pro-inflammatory markers and improved AUC insulin [44].

In Spencer et al. [32] no change in adipocyte size was reported while a significant reduction in macrophage content was found. In our study, mRNA evaluation shows a significant decrease in pro-inflammatory markers raising the question of a potential mechanism altering macrophage function rather than their number. To this end, we assessed the overall changes in gene expression within AT and found, among several differentially expressed genes, for APOE to be highly upregulated. An interesting observation was an increase in MMP9 mRNA levels which challenges the decrease in MMP9 levels previously described in human studies after supplementation with ω-3PUFA [45, 46]. It is unclear whether this may be related to the higher dose and longer duration of supplementation adopted in our study. This finding could be further investigated in future studies. In light of these data, we chose to pursue APOE, which DAVID analysis showed to be present in the top 5 enriched gene pathways. APOE is best known for its role in lipid metabolism [47]; regulating production, conversion, and clearance of lipoproteins [47, 48]. Recent data have described adipose-derived APOE deletion in rodents result in increased insulin sensitivity and decreased AT inflammation [49]. To our knowledge, this is the first human study to identify changes in APOE mRNA expression and plasma protein levels after fish oil supplementation. Due to the controversial role of APOE in metabolism, we sought to determine whether the mRNA changes observed were coming from the adipocyte fraction of our biopsy. Interestingly, our analysis showed no increase in APOE mRNA level after fish oil in pure fractions of adipocytes. This leads us to speculate that the increase we have observed may derive from the stromal vascular fraction containing a mixed cell population. We propose for APOE derived from a non-adipocyte source to decrease inflammation without an apparent change in macrophage number. Clearly, further studies are warranted to elucidate the mechanism(s), function, and source(s) of the elevated APOE levels we report here.

Our study has some limitations. We recognize that this was not a double-blind randomized placebo trial; however, wherever possible, all data acquisition and analysis were conducted blindly. It has been previously noted that the quality of the oil-based placebo may alter inflammation and insulin sensitivity in the control group leading to a smaller difference between treated and placebo groups (reviewed in [19]). While our sample size is sufficient to show biological relevance, a greater number of subjects are needed for further validation before these findings can be translated into clinical recommendations. In addition, greater numbers of study subjects enrolled will allow for evaluation of gender, race, and/or ethnicity effect on our outcomes. Another potential explanation for discordancy among several FO interventional studies may be the impact of genetic heterogeneity on ω-3PUFA responsiveness [50].

Future studies are needed to explore the connection between fish oil and modulation of APOE expression. Larger cohorts are required to confirm our observations which could then be translated into clinical practice.

## Supplementary information

Supplemental file
