# Supplementary material for: ω-3PUFA supplementation ameliorates adipose tissue inflammation and insulin-stimulated glucose disposal in subjects with obesity: a potential role for apolipoprotein E
Source: Int J Obes (Lond). 2021 Mar 22;45(6):1331–41. doi: 10.1038/s41366-021-00801-w (PMC8159741; doi:10.1038/s41366-021-00801-w)

Supplemental information provided as PDF. Fig S1; flowchart describing recruited subjects and reasons for drops or exclusion from protocol. Fig S2; H&E staining of Sc AT before and after ω-3PUFA supplementation. Fig S3; Quantification of macrophage counts before and after ω-3PUFA supplementation. Table S1; primer sequences used for RT-PCR gene analysis. Table S2 displays the results of independent lab analysis of the Nature Made fish oil used in our study.

***Hernandez et al.,* Supplementary information**

**Figure S1.** Participant flow chart.

**TABLE S1** Primer sequences for Sc AT qRT-PCR

| Genes |  | Primer sequences (5' -> 3') |
| --- | --- | --- |
| ACTB | Forward Primer: | AAACTGGAACGGTGAAGGTG |
|  | Reverse Primer: | AGAGAAGTGGGGTGGCTTTT |
| ARG1 | Forward Primer: | GTGGAAACTTGCATGGACAAC |
|  | Reverse Primer: | AATCCTGGCACATCGGGAATC |
| ARG2 | Forward Primer: | CGCGAGTGCATTCCATCCT |
|  | Reverse Primer: | TCCAAAGTCTTTTAGGTGGCAG |
| iNOS | Forward Primer: | AGGGACAAGCCTACCCCTC |
|  | Reverse Primer: | CTCATCTCCCGTCAGTTGGT |
| CD68 | Forward Primer: | GCTACATGGCGGTGGAGTACAA |
|  | Reverse Primer: | ATGATGAGAGGCAGCAAGATGG |
| CD163 | Forward Primer: | CGGCTGCCTCCACCTCTAAGT |
|  | Reverse Primer: | ATGAAGATGCTGGCGTGACA |
| CD206 | Forward Primer: | TTCGGACACCCATCGGAATTT |
|  | Reverse Primer: | CACAAGCGCTGCGTGGAT |
| MCP-1 | Forward Primer: | AGTCTCTGCCGCCCTTCTGTG |
|  | Reverse Primer: | CATCTGGCTGAGCGAGCCC |
| ADIPOQ | Forward Primer: | TGCTGGGAGCTGTTCTACTG |
|  | Reverse Primer: | TACTCCGGTTTCACCGATGTC |
| LEP | Forward Primer: | CACACGCAGTCAGTCTCCTC |
|  | Reverse Primer: | AGGTTCTCCAGGTCGTTGG |
| PPARG | Forward Primer: | GCCGAGAAGGAGAAGCTGTT |
|  | Reverse Primer: | CTCGCCTTTGCTTTGGTCAG |
| CEBPA | Forward Primer: | CCATGCCGGGAGAACTCTAA |
|  | Reverse Primer: | ATGTCGATGGACGTCTCGTG |
| FABP4 | Forward Primer: | ACTGGGCCAGGAATTTGACG |
|  | Reverse Primer: | CTCGTGGAAGTGACGCCTT |
| FASN | Forward Primer: | CTTGCAGGAGTTCTGGGACA |
|  | Reverse Primer: | CCGTCCACGATGGCTTCATA |
| APOE | Forward Primer: | GTTGCTGGTCACATTCCTGG |
|  | Reverse Primer: | GCAGGTAATCCCAAAAGCGAC |
| APOC1 | Forward Primer: | TCCAGTGCCTTGGATAAGCTG |
|  | Reverse Primer: | GGCTGATGAGTTCCCGAGC |
| MMP9 | Forward Primer: | TGTACCGCTATGGTTACACTCG |
|  | Reverse Primer: | GGCAGGGACAGTTGCTTCT |
| MMP7 | Forward Primer: | GAGTGAGCTACAGTGGGAACA |
|  | Reverse Primer: | CTATGACGCGGGAGTTTAACAT |
| MS4A6E | Forward Primer: | TTCTCCTGTCTGTCAACCCG |
|  | Reverse Primer: | GGCTTTGGCTCTATGGCAGT |

**TABLE S2** Nature Made burp-less fish oil product analysis

| Analyte | Unites | Spec. | Results |
| --- | --- | --- | --- |
| Total Fatty Acids | mg/serving | NA | 1330 |
| Total Saturated Fatty Acids | mg/serving | NA | 414 |
| Total Mono-unsaturated Fatty Acids | mg/serving | NA | 332 |
| Total Poly-unsaturated Fatty Acids | mg/serving | NA | 588 |
| Total Omega-3 Fatty Acids | mg/serving | NA | 533 |
| Total Omega-6 Fatty Acids | mg/serving | NA | 55.3 |
| Total Trans Fatty Acids | mg/serving | NA | 5.96 |
| Total EPA & DHA Fatty Acids | mg/serving | NLT 500 | 466 |
| Total Omega-3 Fatty Acids (non EPA/DHA) | mg/serving | NLT 100 | 67 |

**Fig S2.** H&E staining of Sc AT before and after ω-3PUFA supplementation.

Pre-FO

Post-FO


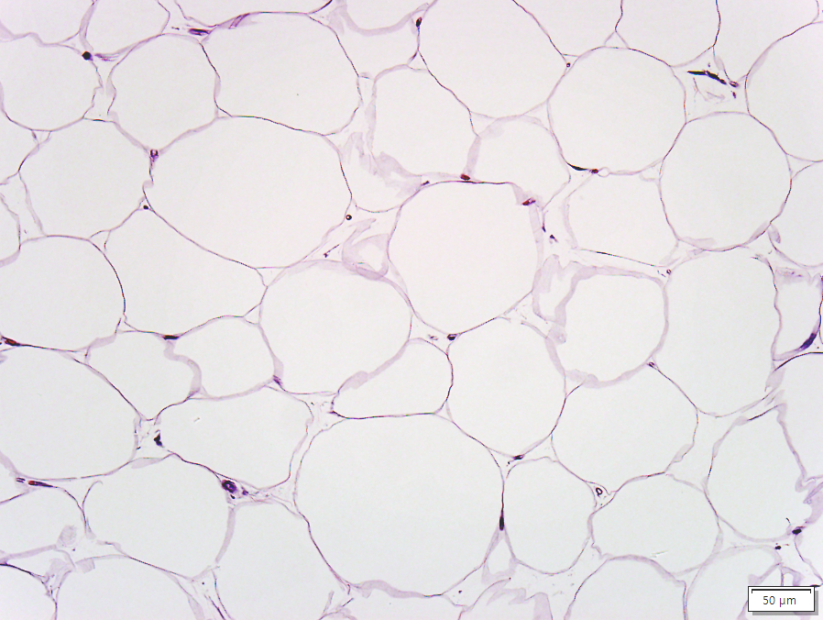

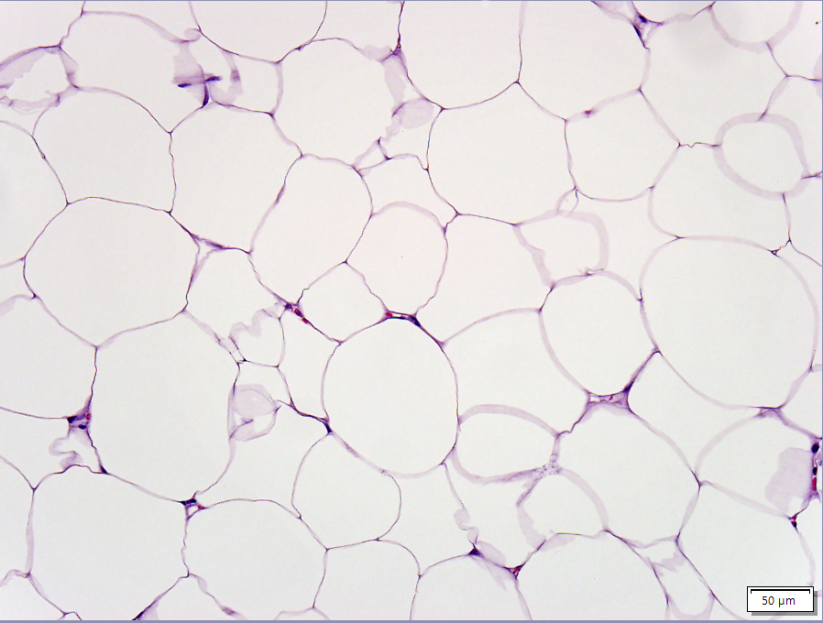


**Fig S3.** Quantification of macrophage counts before and after ω-3PUFA supplementation.


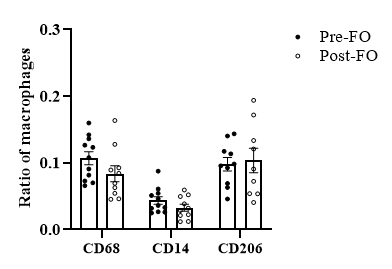

Supplement: Supplementary file 1 — Supplemental file [file 41366_2021_801_MOESM1_ESM.docx]
